# What They Say in Remitting

**Published:** 1910-03

**Authors:** 


### The Journal's Own Page.

### What They Say in Remitting.

"May the new year bring you many new subscribers to that old reliable journal the 'Red Back.' *Homines doctissimi* editor. May he be blessed with the spring of youth for many years to come so that he can inculcate into the medical profession the old-time medical ethics *optimus et maximus.*"—B. F. CALHOUN, Beaumont, Texas.

---

"May you live always."—DR. O. I. HALBERT, Waco, Texas.

---

"I enjoy every number."—W. G. JAMESON, Palestine, Texas.

---

"Keep up the fight for clean medicine, doctor."—F. A. YOUNG, Montgomery, Texas.

---

"Keep up the fight for a free press and freedom of thought for doctors."—W. R. EDWARDS, Devine, Texas.

---

"Congratulations for your bold editorials and best wishes for a prosperous year."—JAS. T. JELKS, Hot Springs, Ark.

---

"Enclosed find check for five dollars. So long as you publish the 'Red Back' just so long send to me."—E. B. PARSONS, Palestine, Texas.

---

A subscriber suggests that Lydston get permission from the Council of Pharmacy to prescribe a bottle of Simmons' Regulator for the Boss of the Octopus.

---

"Camp on the trail of the Octopus until its tentacles slough, and the profession again assumes its pristine purity, honor and dignity." —GEO. F. PERRY, Hamilton, Texas.

---

"Enclosed please find one dollar in good money for the best medical journal in the United States. Let it come and continue to make it hot for those who ought to be in hades."—R. F. HARNESBURGER, Beckville, Texas.

---

"I do not approve of your policy, in ads. carried, but I am going to bear with same for a while, until we can get Simmons out of the chair. What a shame for the medical profession of our country to tolerate such a man, with his past record, at the head of our *Journal*, and at the same time allow him to hold three official positions in the one organization. I am a member of the A. M. A., but am ashamed of the Secretary's record."—L. L. CAHILL, Springer, New Mexico. [The same ads. are carried by all the journals except those controlled by the Simmons gang, and carried by them, until recently.—Our own Chase only got rid of his last year.—D.]

---

"Enclosed you will find one dollar, which will assist you in your manly and noble fight for what is right and just in our medical association. I sincerely hope that you may be able to successfully hold up to view the high and honorable principles which have so long characterized the medical profession. It is to be hoped that you can show to those who will look the great danger which lies ahead, and bring about a mighty effort to keep graft and greed and all the corruptions that go with them from ruining utterly the grandest, the noblest of all professions. I hope for the final triumph of the 'Red Back,' Daniel and Lydston."—J. M. Nicks, M. D., Ranger, Texas.

